# Supplementary material for: Exploring barriers to care home research recruitment during the COVID-19 pandemic: The influence of social media recruitment posts and public sentiment
Source: PLoS One. 2024 Jun 21;19(6):e0303609. doi: 10.1371/journal.pone.0303609 (PMC11192410; doi:10.1371/journal.pone.0303609)
Supplement: Attachment — Submitted filename: CCPONE Reviewers comments and responses May MS.docx [file pone.0303609.s002.docx]

|  | Reviewer #3: comments | Authors responses |
| --- | --- | --- |
| 1. | Abstract :The title and bits of the abstract make it sound as if the care home itself (including residents) are the focus of the research (and the research that this sub-study is situated in) | Thank you for this comment. We have re-worded the title and the abstract to be more precise. New Title:  Exploring barriers to care home research recruitment during the Covid-19 pandemic. The influence of social media recruitment posts and public sentiment. |
| 2. | The paper itself is more about public sentiment to care home staff. | Thank you for your comment. We have explored the influence of public sentiment on research recruitment which is clearly stated in the title and the abstract. |
| 3. | I became confused about the post 1 and post 2, and initially thought it was related to the two aims. | Thank you. In the last paragraph of the introduction, we have added the signposting of the two recruitment posts to clarify this. Also, in methods of the abstract, we highlighted that analysis is from 2 Facebook posts. |
| 4. | Not clear who the participants are in this study. | Thank you. This has now been clarified in the study design section p7.line 125. ‘Made by the public’ |
| 5. | Background –  It becomes clear that this study is situated in a wider study on the mental health and wellbeing of care home staff. This revelation was a bit late as I initially thought it was about recruiting care homes to research (for residents) | Thank you. We have now clarified this in the abstract under methods.  This cross-sectional study analysed comments from two Facebook posts (available June-October 2021) advertising a study on psychological support for care staff during the pandemic. The study was situated within a larger investigation into the mental health and wellbeing of care home staff and employed both qualitative analysis and quantitative methods (word count and correlations between words used and between posts). |
| 6. | Page 5, line 87, you talk about the fact that its difficult to recruit care homes to research. This is true, but there are some good initiatives out there such as the ENRICH network that are worth mentioning. You do talk about this again in your conclusion. | Thank you for this. We agree there are some good initiatives such as ENRIH network, and we have added this information in this section.  ‘…in addition to some UK initiatives such as ENRICH network .’ |
| 7. | Methods – I’m not convinced that you have really done a thematic analysis as considered by Braun and Clark. There are about 200 data items in total. This sounds more like a content analysis than a thematic analysis. Have a look at later writing eg Braun and Clarke 2021. As B&C would say, ‘themes don’t emerge’ | Thank you. We have made some changes to reflect this. We replaced emerged with identified. However, we did not have predefined categories which is a prerequisite for content analysis. We coded, categorised and interpreted the data over the six step approach, which lead us to identifying the themes which fits with thematic analysis. |
| 8. | I think the findings/themes developed are interesting but in order to address what it says in the title, but you need to discuss how these themes act as barriers to recruitment more. | Thank you. We agree with this statement. Explicit connections have now been made between the themes and the research questions. This results in merging of the vaccine and mistrust theme as the issues with vaccine were also linked to mistrust. Changes also made in discussion over the pages 10-13 and 18-20. |
| 9. | In the methods it’s still not clear who the participants are. By the results this has become clearer that it is likely primarily to be members of the UK public (particularly to post 1). I don’t think this is tidy enough. | Thank you. We corrected this.Please see response to point 4 & 5 above for changes. |
| 10. | Don’t you need ethical approval for this kind of research? Or at least some discussion of ethical issues? See this for example. <https://www.gla.ac.uk/media/Media_487729_smxx.pdf> | Thank you. Ethical approval was granted from the Faculty of Health and Life Sciences at Northumbria University REF:32662NU and consent was waived. |
| 11. | I get the sense that this paper was based on getting some interesting reflections back from your recruitment method and thus a bit of an additional publication. | Thank you for your comment. We had an objective to report on social media recruitment due to challenges with recruiting for research during the pandemic. |
| 12. | Results – I thought there was some interesting data in here. The reflections on mistrust of researchers and timing with the required vaccination of English (not Welsh, not sure about Scotland) care home staff to have a COVID-19 vaccination are interesting. | Thank you |
| 13. | The quantitative analysis seems less useful. I’m not sure what the point of comparing +ve and -ve words for post 1 and 2 are, if you don’t know who your respondents are. | Thank you. While it is true we do not know exactly who the respondents are, Linguistic inquiry word count and comparison in public discourse on social media has been used in recent years to help with the understanding or predictability of mood and behaviour models. This can give us an indication/ insight in the understanding of public sentiment in similar situations (recruitment adverts). |
| 14. | I’m not sure the word count analysis aspect added very much to the paper because there is no way to confidently attribute the number of times a word is said with whether it presents as a barrier to recruitment – but this is mentioned in their limitations. | Thank you for your comment. Linguistic inquiry word count in public discourse on social media has been used in recent years to help with the understanding or predictability of mood and behaviour models. This is linked with the locus of control theory, which looks at the extent to which people believe they control their lives.  What this means for barriers to recruitment is that those that feel they have less control over their life, they are more likely to have mistrust towards government and researchers and therefore less likely to participate in research. Thus, as mentioned before, doing work that can increase trust in researchers by building relationships with care homes, can change the perception HCWs have towards researchers. |
| 15. | Discussion and conclusion  I do agree that we need better collaboration between care home staff and researchers. | Thank you for your comment, we appreciate this. |
| 16. | We still don’t know what the results from the survey reported. Was this method of recruitment ultimately successful or not. Your last sentence of the conclusion suggest it was, but we have no evidence of that. | Thank you for your comment. This information is now in the discussion on p18 and just before support. |
| 17. | You reflect on the lack of knowledge of who is responding to the post, but I think this is a more fundamental problem. Those people who reply to facebook adverts with text rather than just a click to ‘find out more’ are more likely to be confrontational, and you know nothing about their background. | Thank you. We agree that that could be the case, hence we have put that in the limitations section. |
| 18. | Do we know if facebook advertising is more successful at recruiting care home staff than any other section of the society? | We found that Facebook advertising for recruitment of care staff was very useful as we used multiple approaches and this approach yielded best results. |
| 19. | Overall style |  |
| 20. | There are a number of lengthy sentences that could be rephrased to be clearer and more concise (e.g., 100-104, study design section, 307, 408-413) and also a number of spelling and grammatical mistakes throughout (e.g., 321, 401) | Thank you. We have reviewed and corrected the named sections. |
| 21. | 21 - first sentence could be rephrased ‘which got’ is weird to read? Maybe ‘and was’ or ‘which was’ | This was changed to ‘was’ |
| 22. | 54 – in what ways? | We are not clear exactly what the question is here, however we have restructured the last sentence there to be clearer. |
| 23. | 64 – ‘were also subject to’  77 – such as? | We are not sure what these re referring to? |
| 24. | 78-79 – repeat of earlier sentence | Thank you for the comment. The two sentences were referring to two different points, but we have added ‘another ‘ to clarify the point. |
| 25. | 100-104 – long sentence | Thank you. We restructured the sentence to read better and clearer. |
| 26. | 107 – this could be phrased more scientifically? | Thank you. We added reference here. |
| 27. | 114 – ‘A cross-sectional, retrospective observational review of comments on specific social media recruitment posts (n=2) was undertaken’. | Thank you. This has been corrected. |
| 28. | Study design – Second sentence is lengthy and could be rephrased | Thank you. This has been broken down and corrected. |
| 29. | 126 – advertised a research opportunity? | Thank you for your comment. We have inserted a research opportunity in the suggested sentence on p.7 |
| 30. | 134 – specify these stages? | Thank you for your comment. We couldn’t understand which stages you wanted us to specify. Nonetheless we tried to clarify the sentence by adding Facebook to the post 1 and post 2 |
| 31. | 151 – correlations were used to explore? Correlations were conducted? | Thank you. This has been amended to ‘correlations were conducted’ |
| 32. | 166 – Most data points relating to this theme highlighted a lack of support – how many? State (n= X) | Thank you. We have added n=20 for the most data points that related to lack of support. |
| 33. | 174 – same as above | Thank you. We have added n=55 |
| 34. | 233 – ‘many levels’ - What other levels? | Thank you. We have clarified this and now reads: Blame was palpable at multiple levels (care home staff, NHS, Government and academia) and on occasions were extreme and expressed as seeking vengeance |
| 35. | ble 3 – is this missing ‘positive emotion words’ in the top row? | Thank you. This is not missing positive emotion in top left corner. All correlations are correctly displayed. |
| 36. | 307 – lengthy sentence | Thank you. This has now been amended with semicolons to break up the sentence.  Negative emotion word use also positively correlated as follows: with words associated with support (r = .27, p = .03); the COVID-19 pandemic (r = .38, p <.001) in post 2; and words associated with vaccines in post 1 (r = .18, p = .04). |
|  | 311 – offers insights into why recruitment to care home studies during the pandemic, using social media, may have been challenging, based on the sentiments contributed by HCWs and by the public | Thank you. We have now amended. Taken together, our findings also offer some insights into why recruitment to care home studies during the pandemic, using social media may have been challenging based on the sentiments shared both by HCWs and by the public. |
|  | 321 – spelling mistake ‘were > where’ | Thank you. This has now been amended to where. |
|  | 322 – meaning? | Thank you. We have added a paragraph to this now on page 19 to clarify. |
|  | 343 – which in this case could mean? | Thank you for this. We have now restructured this paragraph and added additional information. Also, vaccine and mistrust themes were merged, so the discussion reflects this. |
|  | 377 – participants? | Thank you. We have replaced this with ‘respondents’ |
|  | 382 – spelling – limitations | Thank you. We corrected this now |
|  | 401 – capitalised W | Thank you. We couldn’t find where we needed o change and use capital W |
| 37. | 403 – may have been used? | Thank you for your comment. We couldn’t find on the named line what this refers to. |
| 38. | 405 – unsure what this means: ‘should not be considered a one-time input’ | Thank you. We have clarified this now. .. should not be considered as a one – off strategy for recruitment but built in as an integral avenue for recruitment to research with appropriate ethics approval especially with hard to reach groups. |
| 39. | 408-413 – lengthy sentence could be rephrased and split up | Thank you. We have restructured this now. |
| 40. | The influence of social media posts – not sure it shows this as much as public sentiment towards the posts | Thank you. We have said in the limitation the following: Finally, we want to acknowledge that the method used is insufficient to address aim 1 entirely and future qualitative studies should be undertaken to explore this further. Aims are: 1) gain understanding of potential barriers to recruitment of HCWs in UK care homes, and 2) explore public sentiment towards care home research and care homes in the context of the COVID-19 pandemic. |
| 41. | Reviewer #4: comments | Authors responses |
| 42. | * No need to use abbreviations of authors' names in the manuscript (page 6 and elsewhere). The reader does not need to know who did what. | Thank you for your comment. Precise reporting was requested during an earlier round of peer review. We believe the use of abbreviations to precisely convey each authors contribution to the conduct of the study enhances the rigour and trustworthiness of the study. We therefore hope you can understand our decision to leave such reporting unchanged. |
| 43. | * The writing style often lack a terse, technical, academic writing style associated with such manuscripts. Eg. 'This got', 'To use' on page 2 and elsewhere. Later, on p. 7. "after this", 'via discussion'... | Thank you for highlighting instances of where academic writing style could be strengthened and we have amended accordingly throughout. |
| 44. | * By target population (p. 3), are the authors referring to the study's respondents? | We have restructured the sentence . We hope this provides clarity to a naïve reader that we are referring to codesign approaches in the research population rather than study respondents. |
| 45. | *. Lines 97 and 98 on p. 5 need a citation to strengthen the credibility of the claim. | Thank you. We have added the following citation to strengthen this:  Tsao, S. F., Chen, H., Tisseverasinghe, T., Yang, Y., Li, L., & Butt, Z. A. (2021). What social media told us in the time of COVID-19: a scoping review. *The Lancet Digital Health*, *3*(3), e175-e194. |
| 46. | * P. 5, L90 - why don't HCWs have access to work email? The answer may explain the relatively low response rate and quality of the reported findings. | Thank you. Prior research has illustrated this as a potential barrier to study recruitment. However, the explanatory mechanism for this barrier remains underexplored; and yes, we would agree merits further exploration. We have thus restructured relevant sentences. |
| 47. | * Lines 121+122 (p. 6) are vague. | Thank you. We have restructured this line and hope this provides the necessary clarity. |
| 48. | * Lines 332+333 make a bold claim. Were the negative consequences formally diagnosed? How? By what measures? Is there a citation to substantiate this?* | Thank you for highlighting this sentence. We have revised this sentence (and the following sentences) accordingly to make this claim more tentative. However, we believe emergent evidence cited in the follow up sentence supports claims made. |
| 49. | * No formal explanation provided for the low uptake claimed on line 334. | Thank you. We agree that understanding explanatory mechanisms unpicking the low uptake of well-being interventions such as PFA during the COVID-19 pandemic is merited. However, the nascent evidence base to our knowledge has not yet reported explanatory mechanisms for this phenomenon. The need for further research to understand these explanatory mechanisms is reported in the contemporaneous literature ^1^   1. Schoultz M, McGrogan C, Beattie M, Macaden L, Carolan C, Dickens G. Uptake and Effects of Psychological First Aid Training For Healthcare Workers’ Wellbeing in Nursing Homes: A UK National Survey.PLOS One. 2022 Nov 17(11) <https://doi.org/10.1371/journal.pone.0277062> |
| 50. | * Heading on page 18 should read Limitations of The/This Study'. | Thank you for your suggestion. The submission guide is not specific on this . In addition, if we change the heading to limitations only and omit strengths while explicitly discussing strengths and limitations in the section will incorrectly signpost the reader for this section. |
