# Supplementary material for: Exploring barriers to care home research recruitment during the COVID-19 pandemic: The influence of social media recruitment posts and public sentiment
Source: PLoS One. 2024 Jun 21;19(6):e0303609. doi: 10.1371/journal.pone.0303609 (PMC11192410; doi:10.1371/journal.pone.0303609)
Supplement: Attachment — Submitted filename: Plos 1 revision december 2023_MB.docx [file pone.0303609.s003.docx]

| Reviewers comments | Authors responses |
| --- | --- |
| 1. There is no theoretical lens driving the study. The addition of a relevant theoretical lens adds to the credibility of the study, and improves the methodology and findings. | Thank you for your valuable comment. We have incorporated Health Belief Model (HBM) in the manuscript to help contextualize our research within the broader literature and guide the interpretation of our findings. Please see changes on page 4. |
| 2. Grammar is problematic throughout the manuscript. E.g. 'care home' (in the title and elsewhere). Do the authors mean 'home care'? 'Research recruitment' (also in the title). Do the authors mean 'recruitment research'? '... recruitment to home care research...' (p. 3). Etc. | Thank you for your comment.  Regarding the term 'care home,' we acknowledge that terminology can vary, and we understand the potential confusion. In the context of our study, 'care home' refers to residential facilities where individuals, particularly the elderly, receive assisted living and health and social care services. This is widely used term in UK.  Similarly, we note your observation regarding the phrase 'research recruitment.' We intended to convey the process of recruiting participants for a research study. Again, this is widely used terminology in UK. |
| 3. In the abstract, the introduction starts with an unsubstantiated bold claim. Where is the evidence to support this? | Thank you for your comment. The evidence for this is referenced in the main text page 5:  Law E, Ashworth R. Facilitators and Barriers to Research Participation in Care Homes: Thematic Analysis of Interviews with Researchers, Staff, Residents and Residents’ Families. J Long-Term Care [Internet]. 2022 Feb 23 [cited 2022 Jul 21];2022:49–60.  Moore DC, Payne S, Van Den Block L, Ten Koppel M, Szczerbińska K, Froggatt K. Research, recruitment and observational data collection in care homes: Lessons from the PACE study. BMC Res Notes. 2019 Aug 14;12(1). |
| 4. In the same paragraph the authors claim that social media is 'becoming'... No. It's already there. | Thank you. We have changed this to: has increased since Covid-19 |
| 5. The entire aim (on p. 2) of the study rests on the analysis of two social media posts. The authors make do not address the glaring possibility that this may not be a large enough sample size. | Thank you for your comment. We appreciate your concern regarding the sample size and its potential impact on the robustness of our study findings. We recognize the importance of addressing this consideration explicitly.  The focus of our study on the analysis of two social media posts is indeed a limitation, and we have acknowledged the potential impact on the generalizability of our findings. While our intent was to gain valuable insights into public sentiment and potential barriers to recruitment based on these posts, we have made sure the limitation has been discussed explicitly on page 23. |
| 6. How are the findings 'novel'? (p. 3) | Our study makes several contributions to the existing literature. Firstly, we employed a mixed-methods approach, combining thematic analysis with quantitative exploration of word usage. This hybrid approach provides a comprehensive understanding of public sentiment on social media, which goes beyond a purely qualitative or quantitative analysis.  The thematic analysis revealed three prominent themes: support, mistrust, and blame. These themes reflect the complex and multifaceted nature of public discourse during the COVID-19 pandemic, especially in the context of care home workers. Our findings shed light on the nuanced perspectives and emotions expressed by social media users, capturing the depth of their experiences and concerns.  The quantitative analysis, specifically the word count and t-tests, allowed us to quantify and compare the prevalence of certain words and themes between the two Facebook posts. This approach provides a structured and objective dimension to our findings, enhancing the depth of our analysis.  Moreover, the correlations presented in the study offer insights into the relationships between different word categories, revealing potential patterns and connections in the public discourse. This nuanced exploration of language use contributes to a richer understanding of the sentiments expressed in the social media comments.  In summary, the novelty of our findings lies in the integration of qualitative and quantitative methods, the identification of themes that reflect the complexity of public sentiment, and the exploration of word usage patterns that add depth to our understanding. We believe that our study provides a valuable contribution to the field, offering insights into the public discourse surrounding care home workers during the pandemic. |
| 7. The authors reference a government twice in the first paragraph in the introduction. It's not until the next paragraph we find they are referring to the English government. | Thank you for your comment. The introduction specifies UK national governments page 4 9see sentence below). The study was open to whole of UK and therefore its not specifically English government.  *‘Additionally, the perceived lack of support from the UK national governments further heightened the sense of vulnerability among HCWs, with a substantial proportion considering leaving their professions due to the inadequacies of support systems (2). ‘* |
| 8. The authors assert the negative media portrayal of 'care home staff' without an explanation to why this negative media portrayal exists. This is important in understanding the three themes stated in the research. | We acknowledge the importance of addressing the origins of negative media portrayals to enhance the comprehensiveness of our study. In the revised manuscript, we have included a paragraph that explores potential reasons for the negative portrayal of care home staff in the media. The negative media portrayal of care homes during the COVID-19 pandemic can be attributed to several interconnected factors. Heightened mortality rates within care homes, often resulting from the virulent nature of the virus, have become focal points in media coverage, creating a perception of inadequacy in crisis management. Reports on insufficient protective measures, including shortages of essential equipment, and staffing challenges, such as shortages and overwork, contribute to the negative image. Communication issues, both within care homes and between stakeholders, are highlighted, further impacting the perception of care home efficacy. Public and governmental responses, including policies and financial support, are scrutinized, adding another layer to the narrative. Stigmatization of care homes as COVID-19 hotspots and potential sensationalism by the media contribute to a nuanced and often negative portrayal. This complex interplay of factors underscores the multifaceted nature of the media's influence on public perception during the pandemic. Page 23/24. |
| 9. Why are 19-21 year olds 'hard to reach'? How do we know this? | The term 'hard to reach' is commonly used in research literature to describe certain demographic groups or populations that may pose challenges in terms of engagement, participation, or recruitment. In the context of our study, 19-21 is the numbers for the references , as in the statement is supported by references numbered as 19,20 and 21. |
| 10. The authors refer to MS on p. 7. Presumably this is the name of one of the authors. Given that the use of technology is being discussed in this section, MS could be an abbreviation for Microsoft. | Thank you for this. We have added the term ‘the author’ before MS to clarify this on page 7. |
| 11. In one of the more glaring omissions in the reviewed manuscript is the use of constructs, referred to in the manuscript as themes, namely support, mistrust, and blame. Yet none of these are defined in the context of the study. E.g. Support in the health care industry can take several forms, for instance physical support, emotional support, etc. These constructs merit precise definition which assists in better understanding the data analysis. | Thank you for your comment .  We acknowledge the importance of providing clear and precise definitions for key constructs, as it enhances the understanding of the data analysis and contributes to the overall rigor of the study.  In our study, the emergence of support, mistrust, and blame as themes resulted from the inductive analysis of the data. We aimed to capture the richness and diversity of perspectives expressed in the comments, and these themes organically surfaced during the coding process. However, we recognize the importance of clarifying the contextual nuances of these constructs.  Support, for instance, may indeed encompass various dimensions, including physical and emotional support. The absence of specific definitions was intentional to allow for a broad interpretation that reflects the diverse ways in which participants expressed themselves. We acknowledge that interpretations can vary based on context, and different dimensions of support may be more salient in other studies or contexts. We appreciate your thoughtful guidance . |
