# Supplementary material for: Exploring barriers to care home research recruitment during the COVID-19 pandemic: The influence of social media recruitment posts and public sentiment
Source: PLoS One. 2024 Jun 21;19(6):e0303609. doi: 10.1371/journal.pone.0303609 (PMC11192410; doi:10.1371/journal.pone.0303609)
Supplement: Attachment — Submitted filename: PONe reviewers comments April 2024.docx [file pone.0303609.s004.docx]

|  | Reviewer #5: comments | Authors responses |
| --- | --- | --- |
| 1. | 1. the authors have added in the Health Belief Model in the intro without first describing it and how it is relevant. How does it provide a valuable lens? | Thank you for this comment. We have added the following to clarify the lens of HBM  In this context, HCWs' perception of the threat posed by the virus, both to their physical well-being and mental health, aligns with the core tenets of the HBM such as perceived susceptibility, perceived severity, perceived benefits and barriers, cues to action and self-efficacy which combined shape the individuals health beliefs and influence their health-related decision making. |
| 2. | 2. Were the word categories and bank derived from the study team? Did you seek lexicons to use? What are the hypotheses related to first, second, third person pronouns? | Thank you for your valuable feedback and insightful questions regarding our study's methodology and hypotheses. We appreciate the opportunity to address these points in more detail.  Regarding the derivation of word categories and the word bank, they were indeed derived from the findings of our thematic analysis conducted by the study team. We systematically identified recurring themes and concepts from the qualitative data, which informed the creation of word categories for the quantitative analysis. We did not specifically seek external lexicons for this purpose, as our aim was to develop categories tailored to the specific context of our study.  Regarding the hypotheses related to first, second, and third person pronouns, our analysis was guided by the following considerations:  Perceptions of Personal Responsibility: We hypothesized that the frequency of first-person pronouns (e.g., "I," "me," "my") used in the comments would reflect individuals' perceptions of personal responsibility for their actions and behaviours during the pandemic. Higher usage of first-person pronouns may indicate a stronger sense of personal accountability.  Displacement of Responsibility: We expected that the use of second person pronouns (e.g., "you," "your") might suggest attempts to shift responsibility onto others or external factors. This displacement of responsibility could manifest in statements that attribute blame or emphasise external influences on behaviour.  Locus of Control: We anticipated that the frequency of third person pronouns (e.g., "he," "she," "they") could provide insights into individuals' locus of control regarding the pandemic. Higher usage of third person pronouns may suggest a perception that events are influenced by external forces beyond personal control.  These hypotheses guided our quantitative analysis of word usage to explore perceptions of personal responsibility, displacement of responsibility, and locus of control among participants in the context of the pandemic. We have included additional clarification on these points in the revised manuscript. |
| 3. | 3. For a study described as mixed methods, the authors do not describe commonalities or divergence between the 2 methods (except for support which overlaps as a word category and a theme). For example, in the discussion “With attribution to blame, there was a larger range of 356 negative emotion words than positive emotion words.” Where is the analysis to support this? | These are the steps in the analysis we took:  Quantitative Analysis of Word Usage: Quantified the frequency of positive and negative emotion words used in each post. We manually coded the comments to identify and count words associated with positive and negative emotions. This quantitative analysis provided empirical evidence to support the statement that there was a larger range of negative emotion words than positive emotion words in comments attributed to blame.  Comparative Analysis of Themes and Word Categories: Conducted a comparative analysis between the themes identified from the qualitative data and the word categories derived from the quantitative analysis. Looked for commonalities or discrepancies between the themes and word categories to provide a more integrated understanding of the data. For example, we explored how the theme of blame correlated with the frequency of negative emotion words identified in the word categories.  Integration of Qualitative and Quantitative Findings: we Integrated the qualitative findings with the quantitative data to provide a more nuanced interpretation of the results. Discussed how the quantitative analysis of word usage complements or expands upon the qualitative themes identified in the study. For instance, we elaborated on how the quantitative analysis of negative emotion words reinforces the qualitative theme of blame and its implications for understanding public sentiments towards research in care homes during the pandemic |
| 4. | 4. “Findings suggest that care home staff received little psychological support during the pandemic…” Where is the supporting evidence for this in the results? Does the theme of support only refer to psychological support? It sounds like other kind of support are covered under the theme. | Thank you for your insightful feedback and question regarding the evidence supporting the assertion about psychological support for care home staff during the pandemic and the interpretation of the theme of support in our study.  In our analysis, the theme of support encompasses various forms of support, including but not limited to psychological support. While our discussion highlights the potential lack of psychological support for care home staff during the pandemic, we acknowledge that other forms of support, such as logistical support, social support, and access to resources, are also critical for their well-being.  The evidence supporting the assertion about psychological support for care home staff is derived from several sources within our study:  Qualitative Analysis of Themes: The theme of support emerged from our thematic analysis of the comments on social media recruitment posts. While the theme encompasses different types of support, the discussion in the manuscript focuses primarily on the potential lack of psychological support, as indicated by the greater use of negative emotion words in comments from care home staff (post 2) compared to those from the general public (post 1).  Word Count Analysis: Our quantitative analysis of word usage revealed a higher frequency of words associated with support and negative emotions in comments from care home staff (post 2), suggesting a potential lack of psychological support. Additionally, the positive relationship between words associated with support and negative emotive words, as well as first person singular pronouns, in post 2 comments further supports the notion that care home staff perceived a lack of support, which may have contributed to negative emotional experiences.  Literature Review: The assertion is also supported by previous research (citations 11, 25, 26-28) that has documented low uptake and awareness of well-being interventions among healthcare workers, including care home staff, during the pandemic, as well as increased rates of depression, anxiety, and insomnia among this population. |
| 5. | 5. Could you always tell if a response was by a HCW? | Thank you for your question regarding the ability to distinguish responses from healthcare workers (HCWs) within our study.  In our analysis, we did not have direct identifiers for individuals' occupations or roles. Therefore, we were unable to definitively determine whether a response came from an HCW based solely on the content of the comments. We acknowledged this in our limitations. However, we employed several strategies to infer the likelihood of a response being from an HCW:  Contextual Clues: We considered contextual factors such as the topic of discussion and the post being directly addressing HCWs (i.e., healthcare-related issues, experiences in care homes), which may suggest the involvement of HCWs. Comments discussing firsthand experiences in healthcare settings or referencing specific challenges faced by HCWs during the pandemic were likely to be from individuals working in healthcare.  Language and Terminology: We examined the language and terminology used in the comments, including technical medical terminology, professional jargon, or references to specific healthcare procedures or protocols. While not definitive, the presence of such language may indicate that the commenter has a background in healthcare.  Self-Identification: Some commenters may explicitly identify themselves as HCWs or mention their profession or role in their comments. We took these self-identifications into account when analysing the data.  Analysis of Pronoun Use: As mentioned in our study findings, we conducted an analysis of pronoun use, including the frequency of first-person singular pronouns (e.g., "I," "me," "my"), which may reflect personal experiences and perspectives. Higher usage of such pronouns may suggest that the commenter is sharing their own experiences as an HCW. |
| 6. | 6. How does HBM support the findings? There is no mention of it in the discussion. | Thank you for this. We have added to the discussion to address your feedback.  While our analysis primarily focused on themes of support, mistrust, and blame, integrating insights from the Health Belief Model (HBM) enriches our understanding of the underlying mechanisms driving these sentiments. (p17)  Our findings underscore the crucial role of support, encompassing both psychological and logistical assistance, in mitigating the adverse effects of the pandemic on care home staff's well-being. The Health Belief Model provides a valuable framework for interpreting these findings, particularly regarding perceptions of susceptibility, severity, and perceived benefits of seeking support. Perceived susceptibility to negative outcomes, such as mental health challenges, may motivate individuals to seek support services. However, our study suggests that care home staff received little psychological support during the pandemic, aligning with previous research demonstrating low uptake of well-being interventions among HCWs (11,25). This perceived lack of support may exacerbate feelings of vulnerability and contribute to increased depression, anxiety, and insomnia (26-28). The positive relationship between words associated with support and negative emotive words in our word count analysis further underscores the importance of addressing support deficits to alleviate psychological distress among care home staff.(p18-19)  Applying the Health Belief Model, individuals' perceptions of blame may reflect their beliefs about the causes and controllability of adverse outcomes. (p22) |
| 7. | 1. There are several typos throughout, starting in the abstract (extra spaces in line 27, commas in line 28). Others: line 66, 73, 120; grammar in lines 207-208; 386 -missing period; 389 -should be experiences; 472; 479 that should replace than | Thank you for pointing these out. These have happened as a result to tracked changes which have now been corrected and the rest of the manuscript checked for further errors. Some of these lines have moved and are now 210; 405;408;493 and 500. |
| 8. | 2. In the abstract, details about the 2 posts are needed because the results provided have no context. Was it just timing of the posts? Content of the posts? | Thank you for this comment. The following additions provide additional context regarding the timing and rationale behind using social media for recruitment, addressing the reviewer's comment about the need for more details on the context of the two Facebook recruitment posts.  The sentence added to provide context about the surge in social media use during COVID-19 lockdowns: "Social media use has surged since the onset of COVID-19 lockdowns, offering a plausible approach to understanding the barriers to care home research recruitment and gaining insight into public perceptions of care home workers."  The inclusion of the timing of the Facebook recruitment posts: "available June-October 2021" |
| 9. | Define what you mean by ‘choice words’ | Thank you. The term "choice words" typically refers to specific words or phrases chosen deliberately to convey a particular meaning, tone, or emphasis in communication. These words are carefully selected to express one's thoughts, feelings, or intentions in a concise and impactful manner. In the context of analysing comments on social media posts, "choice words" may refer to words or phrases that reflect a particular sentiment, attitude, or opinion expressed by the commenter. These words can provide insights into the commenter's perspective, emotions, or intentions regarding the topic being discussed. |
| 10. | Is ‘with’ missing after co-design in line 55? | Thank you. We added the word ‘with’ after co-design. |
| 11. | What is the limitation of not knowing reach for post 1 (lines 181-2)? Especially related to number of responses to the posts. | The limitation of not knowing the reach for post 1, particularly in relation to the number of responses, is noteworthy. However, it's important to acknowledge that despite the absence of specific reach data for post 1, we believe its reach was likely wider than post 2. This assumption is based on the understanding that post 1 was advertised on the official university page, which typically has a larger and more diverse audience compared to individual or smaller community pages. |
| 12. | References needed for lines 482-502 | Thank you. We have added the following references:    [36](https://journals.sagepub.com/doi/full/10.1177/02692163211017026 36). Tausczik, Yla R., and James W. Pennebaker. "The psychological meaning of words: LIWC and computerized text analysis methods." Journal of language and social psychology 29, no. 1 (2010): 24-54.  37. Kramer, Adam DI, Jamie E. Guillory, and Jeffrey T. Hancock. "Experimental evidence of massive-scale emotional contagion through social networks." Proceedings of the National academy of Sciences of the United States of America 111, no. 24 (2014): 8788.  38. Miller, Edward Alan, Elizabeth Simpson, Pamela Nadash, and Michael Gusmano. "Thrust into the spotlight: COVID-19 focuses media attention on nursing homes." The Journals of Gerontology: Series B 76, no. 4 (2021): e213-e218.  39. Giri, Shamik, Lee Minn Chenn, and Roman Romero-Ortuno. "Nursing homes during the COVID-19 pandemic: a scoping review of challenges and responses." European Geriatric Medicine 12, no. 6 (2021): 1127-1136 |
