# Supplementary material for: Exploring barriers to care home research recruitment during the COVID-19 pandemic: The influence of social media recruitment posts and public sentiment
Source: PLoS One. 2024 Jun 21;19(6):e0303609. doi: 10.1371/journal.pone.0303609 (PMC11192410; doi:10.1371/journal.pone.0303609)
Supplement: Attachment — Submitted filename: PONE-Reviewer comments responses FB comments.Final.docx [file pone.0303609.s001.docx]

Dear Editors and Reviewers

Thank you for your time and suggestions on how to improve the manuscript titled ‘Exploring barriers to recruitment for a care home study during the COVID-19 pandemic: The influence of social media recruitment posts and public sentiment.’

We have addressed all Editors and Reviewers comments and we think the changes significantly improved the manuscript. We have provided responses in the table below to each individual suggestion. Two corrected versions of the manuscript have been submitted. One with highlighted changes to show where the changes were made and one clean version as requested.

We have also adhered to the Journal Requirements below:

1. Please ensure that your manuscript meets PLOS ONE's style requirements, including those for file naming. The PLOS ONE style templates can be found at

https://journals.plos.org/plosone/s/file?id=wjVg/PLOSOne_formatting_sample_main_body.pdf and

<https://journals.plos.org/plosone/s/file?id=ba62/PLOSOne_formatting_sample_title_authors_affiliations.pdf>

Answer. Formating corrected

2. In your Methods section, please include additional information about your dataset and ensure that you have included a statement specifying whether the collection and analysis method complied with the terms and conditions for the source of the data.

Answer: Collection and analysis method complied with the terms and conditions for the source of the data

3. Thank you for stating the following in the Acknowledgments Section of your manuscript:

“We would like to thank the funder RCN Foundation for making this research possible.”

We note that you have provided additional information within the Acknowledgements Section that is not currently declared in your Funding Statement. Please note that funding information should not appear in the Acknowledgments section or other areas of your manuscript. We will only publish funding information present in the Funding Statement section of the online submission form.

Answer: Acknowledgment statement has been removed and replaced with None

Please remove any funding-related text from the manuscript and let us know how you would like to update your Funding Statement. Currently, your Funding Statement reads as follows:

“This study was funded by RCN Foundation.

The award was received by the lead author.

The funder did not play any role in the study design,data collection and analysis, decision to publish, or preparation of the manuscript.”

Please include your amended statements within your cover letter; we will change the online submission form on your behalf.

Answer: The amended statement should state: ”This study was funded by RCN Foundation.The funder did not play any role in the study design,data collection and analysis, decision to publish, or preparation of the manuscript.”

4. Thank you for stating the following in your Competing Interests section:

“None”

Please complete your Competing Interests on the online submission form to state any Competing Interests. If you have no competing interests, please state "The authors have declared that no competing interests exist.", as detailed online in our guide for authors at http://journals.plos.org/plosone/s/submit-now

This information should be included in your cover letter; we will change the online submission form on your behalf.

Answer: "The authors have declared that no competing interests exist.",

5. We note that you have indicated that data from this study are available upon request. PLOS only allows data to be available upon request if there are legal or ethical restrictions on sharing data publicly. For more information on unacceptable data access restrictions, please see <http://journals.plos.org/plosone/s/data-availability#loc-unacceptable-data-access-restrictions>.

Answer: We have made the data available here: <https://osf.io/f7sae/>

In your revised cover letter, please address the following prompts:

a) If there are ethical or legal restrictions on sharing a de-identified data set, please explain them in detail (e.g., data contain potentially sensitive information, data are owned by a third-party organization, etc.) and who has imposed them (e.g., an ethics committee). Please also provide contact information for a data access committee, ethics committee, or other institutional body to which data requests may be sent.

Answer: no restrictions, we have made the anonymus data available

b) If there are no restrictions, please upload the minimal anonymized data set necessary to replicate your study findings as either Supporting Information files or to a stable, public repository and provide us with the relevant URLs, DOIs, or accession numbers. For a list of acceptable repositories, please see http://journals.plos.org/plosone/s/data-availability#loc-recommended-repositories.

We will update your Data Availability statement on your behalf to reflect the information you provide.

Answer: Data is available here: <https://osf.io/f7sae/>

6. Please include your full ethics statement in the ‘Methods’ section of your manuscript file. In your statement, please include the full name of the IRB or ethics committee who approved or waived your study, as well as whether or not you obtained informed written or verbal consent. If consent was waived for your study, please include this information in your statement as well.

Answer: this information was added to the methods section Ethical approval was granted from the Faculty of Health and Life Sciences at Northumbria University REF:32662NU and consent was waived.

| Reviewer 1 | Authors reply |
| --- | --- |
| In the abstract, the rationale for this study should be strengthened along with what additional knowledge this paper will add to the existing literature. The methods are sufficient to address the 1st objective: gain an understanding of potential barriers to recruitment of HCWs in UK care homes | Thank you for your comment.  To strengthen the rationale in the abstract, we rewrote the paragraph:  Recruitment of care home staff to research studies is recognised as challenging. This got further exacerbated by the COVID-19 pandemic and the negative media portrayal during the pandemic. With social media becoming the preferred interaction means during the pandemic it became a suitable approach to understand the barriers to recruitment and gain insight into public perceptions of care home workers.  The discussion and conclusion section highlights the additional (novel) knowledge in the area: Taken together our findings offer novel insights into why recruitment to care home studies during the pandemic including the use of social media might be problematic. |
| Specific comments Abstract: Page 2, lines 21-22 “Recruitment of care home staff to research studies is recognised as challenging, which got further exacerbated by the pandemic”. Please be specific here “COVID-19 Pandemic”. The authors need a sentence to rationalise the study. What additional knowledge this study will add to the existing literature can be highlighted. If space allows, briefly discuss the two Facebook posts here. Under methods, the timeline of review/duration of study is required. | Thank you for your comments. We made the following changes on p2 and p3:  We added the term COVID-19.  To strengthen the rationale in the abstract, we rewrote the paragraph:  Recruitment of care home staff to research studies is recognised as challenging. This got further exacerbated by the COVID-19 pandemic and the negative media portrayal during the pandemic. With social media becoming the preferred interaction means during the pandemic it became a suitable approach to understand the barriers to recruitment and gain insight into public perceptions of care home workers.  The discussion and conclusion section highlights the additional (novel) knowledge in the area: Taken together our findings offer novel insights into why recruitment to care home studies during the pandemic including the use of social media might be problematic.  There was no additional space in the wordcount of the abstract to further discuss the posts here, but this is the information in the methods: Comments were manually extracted from two Facebook posts advertising a study of psychological support for care staff during the pandemic. Comments were analysed qualitatively (thematic analysis) and quantitatively (word count and correlations between words used and between posts).  We added the timeline for the study in the methods: Facebook posts (available between June-October 2021). |
| Introduction Page 5, lines 85-86, “- For example, HCWs often don’t have access to work email or work 86 computer and therefore research information is difficult to distribute.” -need a reference to support this statement AIMS: Aims could be merged with the introduction. It can be the last sentence of the final paragraph under the introduction. If possible, combine the two aims into one. | Thank you for your comment.  We have added now a reference to support this statement on p5.  Beattie, M., Carolan, C., Macaden, L., Maciver, A., Dingwall, L., Macgilleeathain, R., & Schoultz, M. (2022). Care home workers experiences of stress and coping during COVID‐19 pandemic: A mixed methods study. Nursing Open.  On p6 we merged the aims with the background section, but we kept the two separate aims for clarity as they both have a slightly different focus. |
| Methods: The timeline of study is missing here. The authors need to mention the study site/geographical area of the study. The authors need to introduce the data collection team here, along with their background. How did they collect the data? How was the data extracted? Did the team use any validated form of data extraction? The authors also need to clarify how the data were stored and managed. What technology was used to extract data (Manually, AI, etc.) | Thank you for your comment. The following changes were made on p6  Timeline has been highlighted in data collection section: Both posts were live at the same time between June and October 2021.  Geographical data has been highlighted in the same section: Facebook page and set to reach adults in the UK.  Information about data collection and storage has been highlighted in the data collection section p6: Comments were manually extracted on an Microsoft Excel spreadsheet (by CM) from two Facebook posts advertising a study of psychological support for care staff during the pandemic. Data was stored on Northumbria University secure server and only the research team had access to it. |
| Did the study receive any ethics approval? If yes, you need to mention that at the end of the method section. If not, the authors need to clarify , why. | Thank you for your comment.  We have added a section on ethical considerations: Ethical approval was granted from the Faculty of Health and Life Sciences at Northumbria University REF:32662NU and consent was waived. |
| Page 7, line 137 “objectives 1 and 2 were” - There is a difference between the objectives and aims. It would help if you were consistent. How were the codes developed? Did you use any code definitions? Was there any discrepancy among the coders? If yes, how were they minimised? Did you collect data on Emoji? | Thank you for your comment.  On p7 e changed objectives to aim for consistency. Codes were inductively generated with no predefined coding applied. Data were initially independently coded by three researchers with high levels of agreement of initial code generation. Themes were generated via collaborative consensus by the whole team. We have amended the methods section on p7 accordingly.  We did collect data on emoji, however there wasn’t sufficient data to draw meaningful conclusions from it. |
| Results: Page 9, line 157, “199 data points”—What do you mean by that? What about like , share and Emoji? Are they only comments? | Thank you for your comment.  Data points is a wide term and comprises comments and replies. The following has been highlighted on page 9 in the results: 199 data points were collected across the two posts, 133 from post 1 and 66 from post 2. These comprised 90 main comments and linked replies from 94 contributors to post 1, and 26 main comments and linked replies from 38 contributors to post 2. There were 20 shares on post 2, but we didn’t get information on how many shares on post 1. We didn’t consider emoji for analysis as there was insufficient data |
| Anyone can comment on the facebook post. How many of the people who commented were HCWs? How did you differentiate HCWs from the general public? | Thank you for your comment.  We agree that anyone can comment on the post and therefore we highlighted this in the limitation section:  Limitations: Firstly, demographic information could not be collected for those who contributed to the comments. This means controlling for or comparing between variables such as job title (e.g.,HCW v non-HCW), and country (restrictions varied between countries in the UK at different times) was not possible. It is inferred that contributors to the comments on post 2 were more likely to be HCWs due to the content of the comments and the target audience of the page, however similar inferences cannot be made for post 1. |
| Discussion The first paragraph of the discussion section should summarise the findings that address the two study objectives. In the limitation section, the author may acknowledge that the method is insufficient to address objective 1 entirely and recommend future qualitative studies. However, the importance of the data generated from this study should be highlighted. The future use of study findings needs to be discussed. | Thank you for your comment.  We have summarised the results in the first paragraph of the discussion p15:  Four themes emerged from the data namely support, vaccine, mistrust, and blame. There was a greater use of words associated with support and negative emotive words in post 2. Post 2 comments featured significantly more choice words and first-person singular pronouns than post 1. Discussion of mistrust towards researchers was most prominent in post 1. With attribution to blame, there was a larger range of negative emotion words than positive emotion words.  We have highlighted the following in the limitation section: Finally, we want to acknowledge that the method used is insufficient to address aim 1 entirely and future qualitative studies should be undertaken to explore this further. |
| Page 17, line 353 – “the recruitment advert in this post was hosted on the university’s Facebook page and included university branding and reference to the funding organization”. Seems more like a methodology section. | Thank you for your comment.  We have now moved this sentence in the methods section on p6 under data collection and deleted from page 17. |
| Reviewer 2 | Thank you for your comment. |
| Background: Succinct background section with appropriate introduction to the topic. | Thank you for your comment. We appreciate your feedback. |
| - Materials and methods: * An ethics statement is provided in the submission checklist but not within the body of the manuscript. This should be added. I assume a "waiver of consent" was provided? Were participants informed that their posts would be analysed after the fact e.g. a comment on the post advising that their comments would be analysed? * I appreciate the authors use of both qualitative and quantitative analysis- very interesting! | Thank you for your comment.  We have added a section on ethical considerations: Ethical approval was granted from the Faculty of Health and Life Sciences at Northumbria University REF:32662NU and consent was waived.  Thank you for your comment on the analysis, we appreciate it. |
| -Results * Are the authors able to provide any metrics relating to "reach" of the two Facebook posts? They provide figures around number of data points and number of data points. Can they provide further details around potential reach? e.g. how many followers for each page, how many views each post received, reposts etc. *Both social media posts contained a link to a survey. How did the comments/results analysed in this manuscript compare to the findings of the survey study? | Thank you for your comment.  We provided metrics on reach for post 2, but we couldn’t retrieve for post 1. This is now in the results section on p9:  Post 2 reached 14983 people, but we couldn’t retrieve this data for post 1.  We highlighted that support or lack of was consistent with previous findings that show low uptake and lack of awareness of available well-being interventions such as PFA among HCWs (Schoultz et al, 2022) with a reference to the survey for which we were recruiting  . |
| Ethical consideration: have the two Facebook posts since been removed from public view? Once published in the journal, it is possible that a PLOS ONE reader could locate the two posts on Facebook and subsequently identify participants in this study. I appreciate that Facebook users are publicly posting on the two posts and thus should be aware that their comments can be viewed by members of the public. However, all reasonable attempts should be made to deidentify research participants especially considering that they did not provide informed consent for this study. | Thank you for your comment. The comments for post 2 have been removed and we asked the university Facebook coordinator to remove the post from their page. |
